# Understanding of the effect of microbiome on human health: a chemical process engineering perspective

**DOI:** 10.3389/frmbi.2025.1605814

**Published:** 2025-07-11

**Authors:** Xiao Dong Chen

**Affiliations:** Life Quality Engineering Interest Group, School of Chemical Engineering, College of Chemical Engineering, Chemistry and Material Science, Soochow University, Suzhou, China

**Keywords:** chemical process engineering, transport phenomena, human microbiota, biofilm, gut microbes

## Abstract

Aspects about the straightforward linking of gut health or the gut microbiota with existing diseases are critically explored. While there is a popular notion that gut health directly influences overall health and can cause or alleviate diseases, the mechanisms behind these effects are not fully understood. Chemical process engineering (CPE) concepts bring new insights into the effects of human microbiome, which may clarify the fundamental influences. The discussions presented here suggest the future directions of research, which need to be pursued for the benefit of human health.

## Introduction

The human microbiome has emerged as a superhot area of research and development (Wang et al., 2017; Gilbert et al., 2018; Zhao et al., 2018; Chen et al., 2022; Chu et al., 2023; Yu et al., 2023; Liu et al., 2023; Han et al., 2024; Wu et al., 2024; Qian et al., 2024; Chang et al., 2024; da Silva et al., 2024). On the one hand, it is complicated, much like the microbial ecology in other areas, opening to researchers of different levels to conduct studies. On the other hand, it has become a playground for the employment of advanced tools developed in genetics and statistics, which is attractive for multidisciplinary approaches (da Silva et al., 2024). This has become a high-impact publication domain with many medical claims emerging in recent years (Zhao et al., 2018; Yu et al., 2023; Liu et al., 2023; Qian et al., 2024; Chang et al., 2024). Commercially, the attraction of having an effective microbiome medicine to relieve a significant human disease is great, which triggers significant investment, further fueling the interest (Godoy-Vitorino, 2019; Chen et al., 2022; Chu et al., 2023; Liu et al., 2023; da Silva et al., 2024; Ma et al., 2024).

The four prospects for understanding the human microbiome are clinical, microbiological, ecological, and systems (Costello et al., 2012; da Silva et al., 2024). In particular, in a recent comprehensive work on a large number of individuals with varying health conditions, two complete guilds (TCGs) that form a core microbiome structure have been identified (Wu et al., 2024). These two groups of microbes coexist and interact, corresponding to a natural philosophy that at least a two-opponent system should exist to justify a decision. It forms an indicator of health as claimed. The positive group is thought to be beneficial for health. This study is powerful and can have tremendous benefit in helping people label health states.

In any case, the system’s perspective, which could be viewed as encapsulating the engineering principles, involves the top-down and discovery and database independence analysis. However, it has not been elaborated whether the perspective of this system is directly related to the main engineering disciplines, e.g., mechanical, chemical, and electronic engineering. Chemical process engineering (CPE) includes the fundamentals of transport phenomena and reaction engineering, as well as control engineering (Cooper and Jeffreys, 1971; Bailey and Ollis, 1986; Aris, 1989; Levenspiel, 1999; Bird et al., 2002; Chen et al., 2006; Finlayson, 2012; Chen, 2016, 2024). It also specializes in the coupling of these subjects, as well as the process stream effect, for instance, what happens first and what happens next and how the upper stream effect may be propagated down. The recycle and bypass streams are also included. Production survival, storage survival, and digestion survival have been proposed by the author in practice as industry product benchmarks for probiotic delivery since six 6 years back.

Chemical process engineers are involved in studying how the physical and chemical properties of probiotics influence their ability to attach to gut walls compared with the harmful strains. Probiotics ferment dietary fibers and produce beneficial metabolites (e.g., short-chain fatty acids and vitamins) and can be studied as that in fermentation studies in the usual sense. CPE approaches can analyze the metabolic pathways involved, optimizing conditions (such as the pH, concentration, and temperature) to maximize the beneficial output (Chen et al., 2006). Probiotics must survive harsh conditions in the stomach and reach the intestines alive, i.e., the above-mentioned digestion survival. Chemical process engineers have worked on developing encapsulation techniques (e.g., microencapsulation and enteric-coated capsules) to protect probiotics from stomach acids and other digestive fluids, including bile acid, and ensure their release into the intestines (Yao et al., 2019; Vivek et al., 2023; da Silva et al., 2024). Probiotics interact with an individual’s existing gut microbiota, influencing the overall microbial diversity and stability, which is also an engineering interest.

Furthermore, the CPE mathematical models can be developed to simulate these interactions, helping to predict how a probiotic, once introduced, might affect the microbial ecosystem in the gastrointestinal tract (GIT). Another area of CPE is on biofilm formation (Su et al., 2022; Sun et al., 2022). This is well within the area called Fouling and Cleaning, which prevailed as one of the key CPE topics in the past 40 years or so (Muller-Steinhagen et al., 2001; Wilson and Chew, 2010). Probiotics may contribute to the formation of beneficial biofilm, enhancing gut barrier function. CPE studies can focus on how probiotics influence biofilm dynamics and the implications for gut health. This aspect will be more effectively shown in the next section.

Nevertheless, so far, many studies originated by microbiologists or food scientists are works that are mostly along the lines of gut–brain axis and lung–liver axis, among others, not necessarily looking at the dramatic processes in between the microbial gathering spots with the key organs that may be damaged. There are several areas where the CPE can be effective in clarifying the effects of probiotics, from local to body wide, for instance, understanding the mechanisms including competitive exclusion, where probiotics may outcompete harmful bacteria for nutrients in the space of biofilms, preventing pathogenic colonization.

While the health benefits with ingesting probiotics are almost “magical,” as shown in some non-engineering studies, linking the local effect in the gut to disease-specific issues inside the host body seems absent.

Engineering-wise, it is worth mentioning that one of the most fundamental and commonly cited figures in this growing field is the estimate that bacteria residing in the human body outnumber human cells by a factor of 10 or more. This is a striking statement that often serves as the starting point to an introduction into the field. Indeed, if a human being is of a cell population where bacteria take up at least 90% of the body, it is only natural to expect a major role for them in human wellbeing. This has been shown to be an exaggeration, which has been corrected. The microbe number is more likely in the same order as that of the cells, which make up the actual human body (Sender et al., 2016a, 2016b). CPE has been engaged in producing deliverable microorganisms such as probiotics into the GIT so they can survive through the digestive fluids excreted from the body. CPE has also been the basis for understanding the digestion process itself.

CPE has not been employed to look at the mechanisms of probiotic functions in between the GIT and anywhere else inside the body. The two locations are far in between; therefore, an immediate effect may not be possible. A “delay” is necessary, and where the problem may develop within the body needs careful study. The CPE concepts, emphasizing the spatially distributed behavior and the capacity in identifying the transfer processes and reactions that amplify or delay the transfer of the “signals” in between two locations, are argued to be an engineering pillar for the analysis of the probiotic effect in the GIT, the intra-GIT, and the host body. In the following section, the GIT has been illustrated as a series of tubular reactors (Yoo and Chen, 2006; Aguilera, 2018; Chen, 2016; Hernalsteens et al., 2021; Chen, 2024), which leads to a CPE view.

## The GIT is viewed as a series of chemical reactors

The GIT has been recognized as a series of chemical reactors. It may be further recognized as a series of soft-elastic reactors. This recognition has encouraged a stream of *in vitro* experimental work and modeling to be realized (Ferrua and Singh, 2010; Li et al., 2020; Qin et al., 2020; Li and Jin, 2020; Li et al., 2021; Peng et al., 2021; Qin et al., 2022; Sun et al., 2022; Wang et al., 2022; Yang et al., 2024). These works have been carried out to explore greater insights into food digestion and medicine dissolution processes. Furthermore, this recognition has allowed the monumental resources of chemical engineering science, CPE in particular, to be utilized for fruitful multidisciplinary research and development.

Like breathing, where one cannot live without oxygen, one cannot live without the “fuels” extracted from the foods ingested by the GIT to support the “combustion engine” in the host body. The metabolic activities within the body turn biochemical reactions into energy, which powers the processes of any kind in the body, thinking included. The GIT, although seemingly more complex in terms of the material it handles, has its parallel with the respiratory tract (RT).

In the RT, one breathes in air, obtaining the most important component, oxygen, and expels carbon dioxide. It is understood that the exchange of species in RT, particularly where mass exchanges between the airway and the bloodstream, is molecular. It is undesirable for the airway to breathe in particles (PM_2.5_ for instance, and indeed many other sizes) that cannot be degraded and, even if they were, may produce toxic compounds. If the particles become small enough and are somehow absorbed into the body fluids, any accumulation into a sufficiently detrimental amount in an organ would be life-threatening. In recent years, much alarmed by the increased air pollution, one has begun to be seriously concerned about what are actually in the air and which would be more easily transmitted into the lung. In the period during coronavirus disease (COVID) not long ago, this aspect was pushed to a great height. The “flying virus” (small particles) can invade the human body through the RT and initiate immune responses *in situ*, causing fatal outcomes. One may also breathe in undesirable bacteria (pathogenic), noting that the size of free-growing bacteria can be as small as 0.2 μm. It is known that numerous bacterial species from the environment and the host microbiota can migrate and infect the lung tissue, causing pneumonia. The migration of the host microbiota from a CPE viewpoint may occur at the junction of the GIT and the trachea. Deficiencies in specific lung functions and the mucosal immune system lead to increased susceptibility to specific pathogens. In the RT, bacteria can develop adaptive mechanisms to the RT in order to survive in hostile environments related to factors such as co-infecting species and antimicrobial therapies, as well as lung conditions such as inflammatory responses. It has been further noted that, in healthy individuals, there are *Prevotella*, *Streptococcus*, *Veillonella*, *Neisseria*, *Haemophilus*, and *Fusobacterium*, which are the most abundant genera in the lungs (Hilty et al., 2010; Belizário et al., 2023).

When one asks about the benefits of having bacteria in the RT, one may get an answer like the following. Large quantities of bacteria, including Firmicutes, Actinobacteria, and Bacteroidetes, colonize the surface of the respiratory mucosa of healthy people. They interact and coexist with the local mucosal immune system of the human airway, maintaining the immune stability and balance of the respiratory system. It may then suggest that the existence of the bacterial population in the RT, at best, would be for the maintenance of its normal function, which should be mainly for trafficking gases, some fresh in and some waste out as the products of metabolic activities from the inner body. If the bacterial population is more toward the harmful ones, causing inflammation for instance, it would disrupt the lung function, causing more serious outcomes. These may also be due to their excretions that damage the RT norm. One could then say that this bacterium is the cause and the sickness is the effect of it, while at the same time some others may act as the guardians. The exposed surface area inside the lung is huge in response to the diluted state of gaseous molecules.

Since bacteria X can cause damages in the lung, one may argue that there would be good bacteria Y whose function is to protect. Probiotics that boost the RT health of the host have recently gained attention. Clinical studies have revealed that probiotics can improve the immune function and reduce the severity and incidence of lung diseases. All sounds familiar, does it not? On the other hand, unlike the huge attention gained by matters of the GIT, it seems no one has suggested that the RT microbiota is a second or a third brain for the host. Of course, the GIT effect appears more powerful, for instance, the whole business of serotonin generation and control, among others, which is responsible for a large part of the gut–brain axis. Existing physiological processes, such as menstrual cycles, do affect the feeling of the gut, which is a result of the effect on gut functions.

The GIT “breathes” in significantly more complex exterior substances. It ingests materials of high solids, e.g., foods. Along the tract, there is a spatially distributed behavior, digestive and absorptive, and finally fermentative, i.e., a series of chemical reactors. The purposes of these, by natural logic, should be geared toward the extraction of useful molecular species derived from the foods to support the host. The fermentative processes should happen largely in the colonic or the large intestine area as the “last attempt” to convert, through longer residence time periods, the beneficial stuff from the remaining food (food residue). The absorptive processes occur at the most parts of the boundary of the GIT, including the colon. This is mainly through active absorption, while passive absorption coexists in a smaller fraction. The digestive fluids are excreted into the GIT at specific spots, which are in active response to the material being processed in the GIT but controlled by the nerve system. The process must be aligned with the control engineering within CPE. How these rates may be controlled is, however, not known mechanistically and quantitatively.

**Figure 1:**
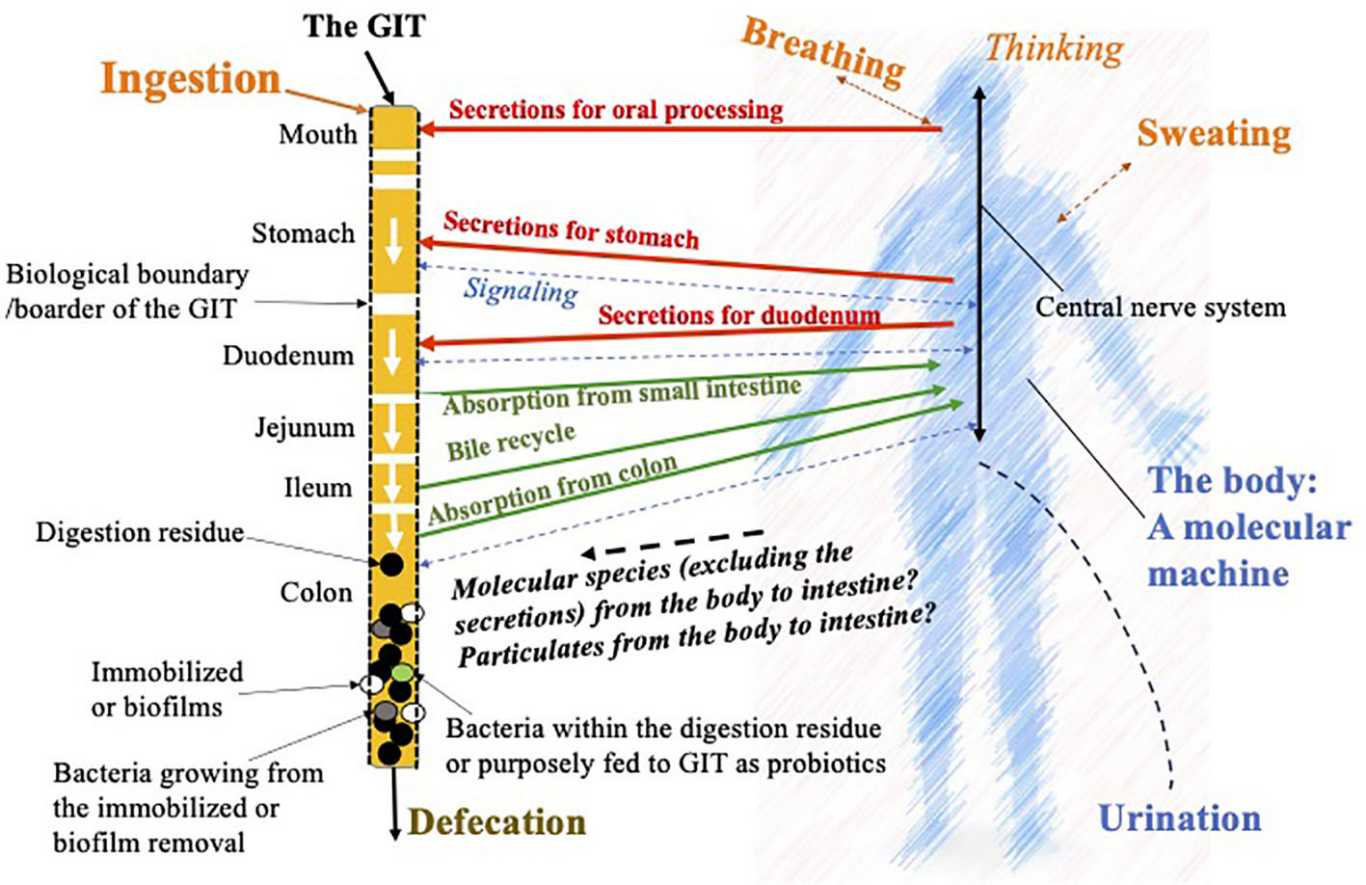
Illustration of the chemical process engineering (CPE) approach to dissect the functions and the linkages to and from the human gastrointestinal tract (GIT) with the host body. This is based on the idea of chemical reaction engineering analysis on nutrition by the author (Chen, 2024), but focusing on the microbial effects in the gut and how they may have further affects anywhere inside the host body.

As illustrated in **Figure 1**, which is derived from earlier arguments by the current author (Chen, 2024), on the left-hand side, the GIT has been singled out so that the spatially distributed behavior of each of the reactors, i.e., mouth, stomach, duodenum, jejunum, ileum, and colon, is highlighted. For the purpose of applying CPE, the GIT is shown as a reactor in series “outside the host body.” The interactions between the host body and the GIT can then be emphasized. Signals obtained through the receptors along the GIT are transmitted to the central nervous system on the right-hand side and back to the GIT for controlling secretions, motility and the like. It is expected that any congestion or discomfort, like a pain, will be processed in this way as well. These may be felt not only at the biological boundary of the GIT but also through the tissue connected with it. Due to the central location that the GIT occupies, the discomfort is felt more significantly.

In **Figure 1**, it is noted that the body feeds secretions, i.e., the biochemicals and the chemicals, which are needed to initiate and accelerate the digestion processes. The undigested materials (food residue) and a few percentages of the remaining bile are sent into the colon. The food residue is further digested through fermentation, which is hoped to create biochemicals and chemicals beneficial to the host body. Here, two parts of the bacteria come to play: one is from the colonies already on the “walls” of the colon, which in CPE terms is often called the biofilm. What is created routinely from the biofilm and get absorbed by the body is interesting to start with, nutritionally and medically. In reality, not all of the species created through fermentation are advantageous. Some are toxic, which may cause local inflammation, or some are even cancerous. In normal circumstances, one would expect that the population and the types of bacterial strains are “reasonably good or normal” so that the majority of people can have pleasant experiences with food ingestion and the GIT. The bacteria that come with the food residue, some of which may be purposefully fed probiotics, together with that “emitted or grown out” from the biofilm, help fermentation in the bulk of the food residue forming feces, which is not expected to cause discomfort such as constipation. Here, the GIT is taken as a reactor that is intended to process food, drink, and medicine, therefore the digested material releasing molecules that can be absorbed into the host body fluids maintaining or improving the host health. Chen (2024) proposed a term called “molecular machine” to describe the processes that go on in the host body (shown in **Figure 1**). The host body, excluding the GIT, cannot produce a solid waste. If somehow some larger particles are contained in the bloodstream, which cannot be discharged through the kidney, they have to find their way back to the GIT from channels including the bile duct or the pancreas, which may cause fouling or scaling there. They may cause malfunctions in the liver or the pancreas, respectively. This may, in part, quietly explain the lung–liver axis for particulate pollutions, such as breathing in PM_2.5_ particles, eventually leading to a liver problem. In comparison, one can see that the RT has little or probably no solid discharge capacity, while the GIT has a great solid discharge capacity. The *in* and *out* mechanisms of the RT and the GIT, respectively, are extremely different, which are worth capturing in order to improve our fundamental understanding of the human body.

**Figure 2:**
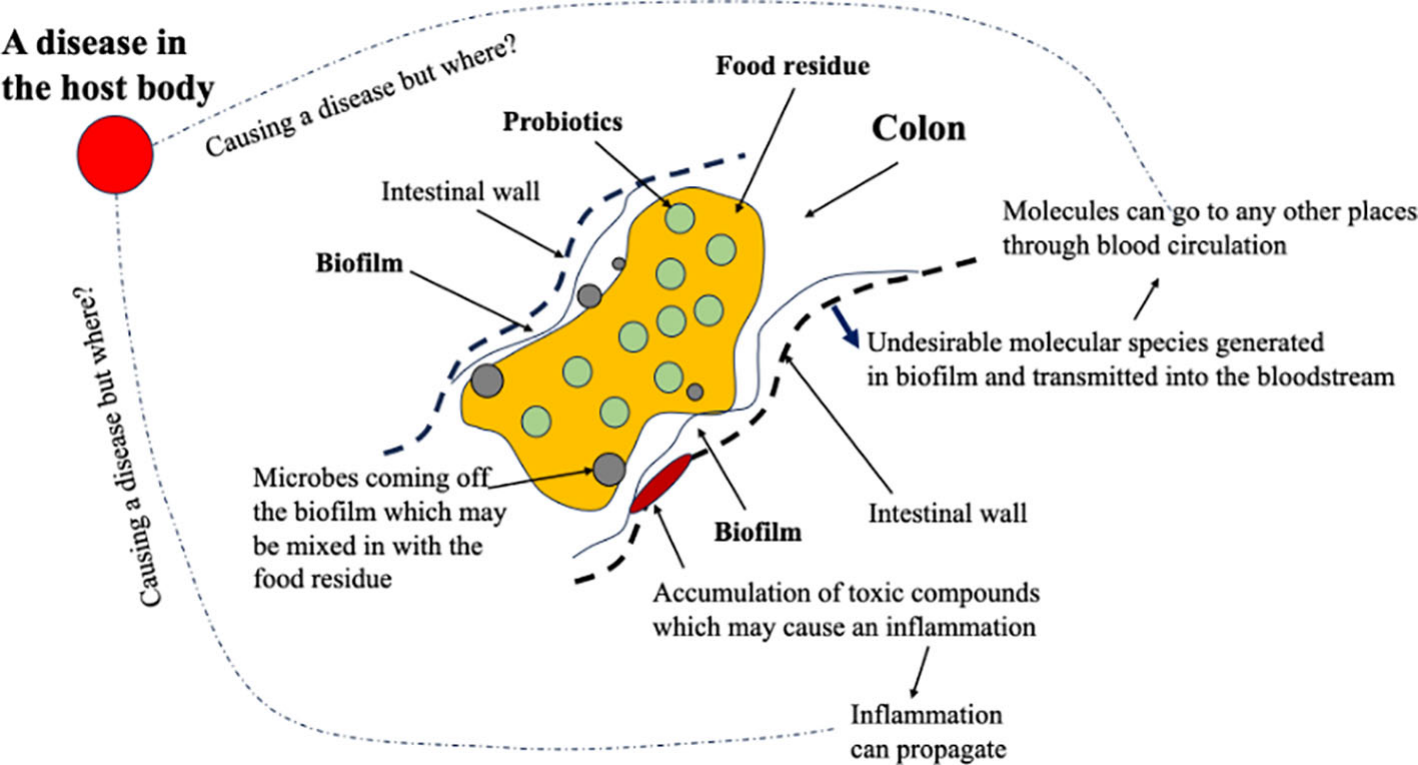
A chemical process engineering (CPE) view of what is going on in a colonic section when the food residue, the probiotics in the food residue, and the bacteria from a biofilm interact. The biofilm may cause local inflammation, the effects of which can propagate. The toxic molecules generated in the biofilm, if any, can be transmitted into the bloodstream, which may gather somewhere in the body, causing a disease.

As illustrated in **Figure 2**, considering the functions of the GIT, with the bacterial populations, in the colon in particular, how they may cause a disease in the host body would have to be through the undesirable molecular species released from the biofilm or from normal cell damages occurring at the base of the biofilm, i.e., a local inflammation. There is a pH distribution in the GIT, which affects the survival of the bacteria in the regions or those traveling through the regions. In the stomach, the pH is approximately 1.0–2.0, in the duodenum 6.1, in the middle small intestine 7.1, in the distal small intestine 7.5, and in the cecum and rectum 6 and 7.0, respectively (Yamamura et al., 2023). Often, probiotics have to be encapsulated to avoid premature death before reaching the location they are supposed to aim at delivering (which is called *digestion survival*; XD Chen, 2019–2025, plenary/keynote presentations worldwide). Biofilms are common in the gut (Jandl et al., 2024). These biofilms are complex structures formed by a number of microorganisms, primarily bacteria, which are embedded in an extracellular matrix (Donlan, 2002; Buret and Allain, 2023). The matrix consists of water and biopolymers such as polysaccharides, proteins, lipids, and extracellular DNA (Buret and Allain, 2023). The “fermentation” in the biofilms can produce a range of chemicals including toxins (Miller et al., 2021; Pop et al., 2022). The gut microbial metabolites such as short-chain fatty acids, defensins, cathelicidins, and lactoferrin can be found in feces (Yamamura et al., 2023). Disruptions to the equilibrium between these biofilms and the host may create invasive pathobionts from these commensal communities and contribute to disease pathogenesis (Buret and Allain, 2023). Being able to adjust or to maintain the populations in the biofilm, which can carry out the normal fermentative processes that do not cause undesirables, must be the key for gut health. These undesirables include those that adversely affect the integrity of the walls of the intestines, creating compromises and even leakages, or those that can, in molecular forms, infuse through the walls and are then taken up by the body fluids to be circulated in the body. Through these mechanisms, the unwell gut health can cause further sicknesses inside the host body, which is away from the gut. In other words, it is noted, once again, that the bacteria cannot themselves transmit through the biological boundary of the GIT, unless there is a compromise or a leak. Any inflammation at the boundary can lead to a local source of antibiotic behavior, which comes through the blood flows. The long-term release of toxic molecular compounds from an “unwell” biofilm, once it has gone into the bloodstream, would be like entering a superhighway that can go to anywhere in the body. This may degrade the health of the host over time and cause a chronic problem.

With a supply of good bacteria that can help adjust the composition of the biofilm, particularly when it is unwell, the inflammation site underneath the biofilm and at the intestinal wall may be reduced through a competitive process. How quickly and to what extent a positive effect can take place are CPE questions. There is a spatial–structural issue with any biofilm; therefore, effective maintenance and/or improvement of a biofilm needs to be studied. However, so far, this seems not currently on the topical agenda of the gut microbiome.

An existing biofilm is generally difficult to remove, even in a chemical industry with harsh chemicals. A large number of studies have concentrated on the pathogenic nature of biofilms, singling out the key bacteria that are detrimental, are available, but not in the context of linking them to the internal diseases. The effect of a probiotic or a cocktail of probiotics would be a slow one, if considered as a medicine. Large doses are expected if wanting to change the situation in a shorter time frame. To start with, the ecology of a biofilm is complex; therefore, identifying the good and the bad becomes difficult, and replacing a biofilm with another one must be more difficult, despite the recent prominent work by Wu et al. (2024) that appeared to have identified conclusively the two main competing bacterial groups in the human gut: one is good and one is bad, which may “fight” over each other to impact on the gut health.

The few percentages of the bile salts that go into the colon may serve to restrain the colonic bacteria from uncontrolled growth or selectively constrain certain microbe populations. From a CPE viewpoint, again illustrated in **Figure 2**, there is no immediate link from the gut bacteria to a disease within the body. A mass transfer pathway needs to be identified. A notable disease may occur where the toxic compounds could gather or stay for longer times. One, as expected, tends to focus on the prime organs such as the liver, pancreas, kidney, and the lung. The flow pathway in each of them may be so characteristic, which makes it easier to “gather” the toxics. With an infection at the biological boundary of the GIT, per se, in the colon in particular, it may take some time to infect anywhere within the host. However, the discomfort felt in the GIT or at the GIT boundary, when lasting for a long time, say days, would cause some responses from the nervous system, causing mental issues such as the development of depression.

A serious disease can be said to be caused roughly by gut bacteria. It starts from the biofilm in the gut (Zhao et al., 2023). Local inflammation may only be of a local disease, colonic infection, or even a cancer. Long-range disease-causing behavior may be through the toxic molecular compounds produced by the biofilm that are transmitted into the bloodstream. Indeed, any fermentative processes in the gut, the colon mainly, which can generate molecules that pass the boundary into the bloodstream. The bloodstream is the superhighway within the host body. In fact, many but single identified diseases inside the host body can be relieved somewhat by altering the patients’ microbiota through adjusting the biofilm productivity and the bulk fermentation of the food residue, reducing the toxic levels in the bloodstream. Although previous works on the delivery of probiotics may be useful, they cannot elaborate on the mechanisms except to expect an improvement of the biofilm. Only when the structure and the composition of the biofilm, including what molecular species it can generate, can be thoroughly studied would a proper understanding of the effectiveness of any probiotics be obtained. The species generated would be extremely important. Other CPE-related questions that appear to not have been mentioned before are: how does an existing biofilm interact with the added probiotics? If the nutrient is already consumed more by the added probiotics, might the biofilm be starved? If there is little feed left after the small intestine, as in much less dietary fibers in the food in the first place, and much less duty needs to be performed, thus the biofilm may be starved too? Should the biofilm be minimized if there is not much food residue in the first place, for instance if the food is so refined that it can supply all the body needs to stay healthy, but produce little residue to go into the colon?

## A simple mass balancing argument based on CPE

To illustrate the CPE perspective, the mass conservation for the food for microbes is given here. Taking an isolated lump of food residue (or a developing feces), which contains “foods for microbes” (FFMs) that are sustaining the probiotics that come with (and within) the residue (assuming a few probiotics are attached on the colon wall or on the biofilm), the biofilm that is in contact with the lump of food residue hardly gives off cells from it, for simplicity, but the FFMs can be transferred into the biofilm surface to feed the bacteria there. Both the probiotics and the bacteria in the biofilm produce molecular compounds that can be transmitted into the bloodstream. There are transfer coefficients for these transmissions according to the mass transfer theories already established in CPE. Assuming that the existing biofilm is the one that produces more toxic molecules that affect the host health gradually and that the probiotics do not, the existence of probiotics in large numbers would consume the available FFMs, thus restricting the productivity of the biofilm, achieving a benefit. Moreover, it is possible that the compounds produced by certain probiotics may be detrimental or limiting to the maintenance of the undesirable cells in the biofilm; this would also achieve a benefit.

If there are no probiotics to compete with the biofilm on FFMs, the types of FFMs must become an interesting topic as they would encourage the biofilm to grow well to different extents. They may make the situation better or worse depending on which kind of microbes they end up promoting preferentially. Indeed, what the probiotics produce during the fermentative actions in the food residue has not been a topic of intensive research.

More complicated scenarios than the above can be panned out and discussed. The simple example here is a fruit for thought encouraging the CPE-informed series of studies on the mechanisms. A more complex scenario is probably how to take probiotics, such as taking probiotics when fasting, before a meal, with a meal, or after a meal, in order to be more effective.

While for many hundreds of years our foods have been refined, good or bad is often the focus of the arguments in nutrition. What about the influence of the refinements on the effects of microbes? Little is known. The CPE approach may help more balanced studies to be carried out and also help quantify the issues more systematically than before.

## Concluding remarks

As its purpose, the GIT is expected to transform the particulate materials orally taken into the GIT into molecular species that are hopefully beneficial chemicals and biochemicals to run the life of the host body, which is a “molecular machine.” The current approach is intended to separate the domains of interest and to look at the continuous but spatially distributed processes in the GIT. The GIT is a series of chemical reactors. As it is intimately connected, through molecular channels and sensors (or receptors), any pains and congestions at the location of the GIT, as it is of a central location over a large part of the host body (geometrically), would cause major responses felt by the person concerned, emotional and psychological responses included as it is a sensory hub. When there is congestion or discomfort in the GIT, it can lead to pain that is felt throughout the body. From the CPE viewpoint, what is going on in the GIT is a reactor in series, and the task of the microbiota is mainly to ferment the food materials as one of the digestive processes, to generate molecules (hopefully good for the body) to supply to be transferred into the host body. How the human microbiota in the GIT can initiate a disease inside the human body is a process that is not fully understood; that is, what is happening in between is less known. For an adverse effect to take place, CPE needs an explanation for what the components of concern are and how these components exert a local affect and be transferred into the body that can trigger an internal problem. The molecules generated from microbial activities, once they have entered the body, may participate in biochemical reactions that could have an adverse effect. The bacteria cannot directly enter the bloodstream; infections at the GIT boundary can cause inflammation. This inflammation might trigger the immune system, leading to antibiotic responses in the body. Chronically, if a toxic condition is sustained, a disease is expected to occur.

The CPE would emphasize the understanding on how the biofilms are structured locally and how they may be replaced or removed like those in the fouling and cleaning area. Biofilms in the GIT are structured communities of microorganisms that can adhere to gut surfaces. They can protect pathogens from the immune system and antibiotics, complicating infections and treatment. Understanding how biofilms can be disrupted or removed is crucial for the development of strategies to restore gut health, particularly in the context of probiotic treatments. It is suggested that what happens in the GIT side, what are transferred into the bloodstream for circulation in the body, and then what and where a disease might be triggered need to be studied.

CPE requires understanding of the processes in between rather than trying to connect what happens in the GIT “directly” to a disease in the body. From a CPE viewpoint, the GIT can be likened to a series of reactors. The microbiota ferments food materials, generating beneficial molecules that are absorbed and utilized by the body. However, the mechanisms by which the gut microbiota can initiate diseases remain poorly understood. To sum up, the key areas of focus for CPE include: identifying microbial by-products that may lead to adverse health effects either from the biofilm or from the probiotics chosen (Sanders et al., 2010); understanding how these components impact local and systemic health; and while bacteria typically do not enter the bloodstream, infections at the GIT boundary can cause inflammation, which may activate the immune system and provoke antibiotic responses. Chronic exposure to harmful chemicals can lead to disease. The “bridge” is the biofilms in the GIT that comprise structured communities of microorganisms that adhere to gut surfaces. These biofilms can protect pathogens from the immune system and antibiotics, complicating treatment. Understanding how to improve the existing biofilms and to disrupt or remove harmful biofilms is essential for maintaining or restoring gut health, particularly with regard to probiotic interventions. Finally, from a CPE viewpoint, the competition between probiotics and the biofilm about the “foods” for them *in situ* of the gut, the colon in particular, may be very important. In fact, from the same viewpoint, if a special FFM can be delivered into the biofilm, when no probiotics are involved, which can actually increase certain bacterial populations while limiting the maintenance of others, this may be a good option for practical purposes (Wang et al., 2023).

Based on CPE, one may also raise questions that are controversial at the moment. For instance, with the use of food fibers, exactly what are their characteristics that can reduce the harm done by a biofilm in the first place, or do they help a biofilm that constantly produces toxic compounds that “slowly poison” the host, eventually causing diseases? Is our life span limited by what the microbiota releases? Based on the above discussions, one may develop more systematic strategies for health promotion and disease prevention through dietary and microbiome-based interventions, firstly aiming for a healthy gut and then aiming for a healthy body.

## Data Availability

The original contributions presented in the study are included in the article/supplementary material. Further inquiries can be directed to the corresponding author.
